# Insomnia in Relation to Academic Performance, Self-Reported Health, Physical Activity, and Substance Use Among Adolescents

**DOI:** 10.3390/ijerph17176433

**Published:** 2020-09-03

**Authors:** Gita Hedin, Annika Norell-Clarke, Peter Hagell, Hanne Tønnesen, Albert Westergren, Pernilla Garmy

**Affiliations:** 1 Faculty of Health Sciences, Kristianstad University, SE-291 88 Kristianstad, Sweden; annika.norell_clarke@hkr.se (A.N.-C.); peter.hagell@hkr.se (P.H.); albert.westergren@hkr.se (A.W.); pernilla.garmy@hkr.se (P.G.); 2 Clinical Health Promotion Centre, WHO-CC, Department of Health Sciences, Faculty of Medicine, Lund University, SE-221 85 Lund, Sweden; hanne.tonnesen@med.lu.se; 3 Department of Social and Psychological Studies, Karlstad University, SE-651 88 Karlstad, Sweden; 4 Health-Promoting Complex Interventions, Department of Health Sciences, Lund University, SE-221 85 Lund, Sweden

**Keywords:** adolescents, alcohol, cigarettes, insomnia, MISS, physical activity, self-reported health

## Abstract

Purpose: Insomnia affects up to one in four adolescents and has been shown to have a negative impact on their mental and physical health. This study aimed to investigate the association between insomnia, academic performance, self-reported health, physical activity, school start time, and substance use among adolescents. Methods: A survey with a cross-sectional design was completed by adolescents (15–17 years old; n = 1504) in southern Sweden. The Minimal Insomnia Symptoms Scale (MISS) was used to operationalize insomnia. A multiple logistic regression analysis was used to analyze the relationship between insomnia and self-reported health, failed school courses, substance use, school start time, family financial situation, screen time, and gender. Results: Insomnia (MISS ≥ 6) was associated with poor self-reported health (OR: 4.35), failed school courses (OR: 1.47), and use of alcohol and/or cigarettes (OR: 1.43). When the combined effect of self-reported health and physical activity were investigated, a combination of low physical activity (≤1 time/week) and poor self-reported health was strongly associated with insomnia (OR: 18.87). Conclusions: Insomnia was associated with other problems that in themselves are risk factors for poor health. This highlights the need for a holistic health-promoting approach to prevent insomnia, such as efforts to promote physical activity, school success, and the reduction of alcohol/cigarette use.

## 1. Introduction

### 1.1. Insomnia

Insomnia is a condition primarily manifested by difficulties initiating sleep, difficulties maintaining sleep, or waking up too early [1]. The prevalence of sleep problems among school-aged children (aged 11–15) in Europe and Canada was 28% among girls and 18% among boys in the Health Behaviour in School-aged Children (HBSC), a World Health Organization (WHO) collaborative cross-national study with 220,000 children and adolescents [2]. A study from the US among 1014 adolescents (13–16 years) showed that 10% of the adolescents fulfilled the diagnostic criteria of insomnia, however, 33% were frustrated with the sleep and had insomnia symptoms [3]. The prevalence of insomnia among adults in Sweden ranges from 12% to 25% [4,5], while the prevalence of insomnia among adolescents in Sweden is approximately 7–24% [6]. Insomnia can lead to other problems for the individual, such as fatigue, distress, and cognitive impairment during the day [7]. Insomnia can lead to health problems over time [8].

### 1.2. Adolescents and Insomnia

Insomnia and insufficient sleep are serious public health concerns that can impact adolescents’ mental and physical health [9]. Adolescence is a vulnerable period that is often linked to the emergence of emotional and behavioral difficulties that impact sleep quality and quantity [10,11]. Sufficient sleep is an important indicator of health among adolescents [12]. A study from Sweden reported that poor sleep has consequences for the daytime functioning of youths (aged 16–20 years), and that severe symptoms of insomnia were related to a higher risk of school absenteeism [6]. Population-based studies indicate that approximately 25–35% of adolescents get insufficient sleep; alarmingly, these estimates are growing [13]. 

### 1.3. Health Habits and Contextual Factors

Healthy habits are important for promoting later-life mental and physical health [14], including sleep quality. For example, a Swiss study among 1581 adolescents and young adults (aged 16–25 years) showed that physical activity affects sleep and is a strong predictor of good sleep [11]. A Canadian study of 5560 children aged 10–11 years reported that a higher physical activity level was associated with longer (10 h) sleep duration [15]. Poor sleep was found to be a predictor of substance use among adolescents [16], and failed school courses were found to be more common in adolescents who slept less than seven hours during a night before school [17].

In addition to habits, there are also contextual factors that are beyond the adolescents’ control that can also be associated with adolescents’ health, such as their family’s financial situation and their school’s start time. For example, the family’s financial situation is correlated to the frequency and type of physical activity performed by adolescents [18].

### 1.4. Aim

Adolescents are in a tumultuous period in their lives characterized by frequent and at times rapid changes in habits, behaviors, and motivations. Further, in the current digital era with rapid societal changes, insomnia is an increasing public health concern. Therefore, further studies are required on factors associated with insomnia. This study aimed to investigate the association between insomnia, academic performance, self-reported health, physical activity, school start time, and substance use among 15–17-year-old Swedish adolescents. 

## 2. Methods

### 2.1. Study Design

A cross-sectional design was used. The study was approved by the Regional Ethical Review Board in Lund, Sweden (EPN 2017/600). All procedures were conducted in accordance with the Declaration of Helsinki [1].

### 2.2. Sample and Data Collection 

Data were collected from four upper secondary schools (three public and one private) in southern Sweden. The participating students (15–17 years old) came from both urban and rural areas. After approval from the school’s administration, written information about the study and its voluntary nature was distributed to students and their parents or legal guardians. All first-year students in these schools were invited to participate in the study. Of the four schools’ 2089 students, 1504 (72%) chose to participate. The web-based questionnaire was distributed in class during school hours between October 2017 and May 2019. 

### 2.3. Instruments

The survey included questions about their gender, perceived family financial situation (5-point Likert scale ranging from “very well” to “very bad”), self-reported health (5-point Likert scale ranging from “very well” to “very bad”), school start time, physical activity frequency (7-point Likert scale ranging from “never” to “≥4 times/week”), failed school courses (4-point Likert scale ranging from “none” to “≥5 courses”), alcohol use frequency (6-point Likert scale ranging from “never” to “≥1 times/day”), cigarette use frequency (6-point Likert scale ranging from “never” to “≥1 times/day”), time spent on social media/gaming (hours and minutes), and time spent watching TV/films (hours and minutes). 

In addition, the questionnaire included the Minimal Insomnia Symptoms Scale (MISS), which consists of three items representing major symptoms of insomnia: difficulty falling asleep, night awakenings, and unrefreshing sleep [19]. Each item has five response categories: no, minor, moderate, severe, and very severe problems, which are scored 0–4, respectively. This yields a total score ranging between 0 and 12, with higher scores indicating more severe insomnia symptoms. The cut-off score of ≥6, previously suggested for identifying insomnia in the general adult population between 20 and 64 years of age [20], was used in this study. 

### 2.4. Data Analysis

Data were first analyzed descriptively as frequencies (n) and percentages (%). The variables “time spent on social media/gaming” and “time spent on TV/films” were merged into a new variable termed “screen time”. Bivariate analysis (chi-square tests) was used to investigate the association between the dependent variable of insomnia (MISS ≥ 6) and the following independent, dichotomized variables: sex (female = 0; male = 1), self-reported health (very well/good = 0; neither good nor bad/quite bad/very bad = 1), failed school courses (none = 0; ≥;1 = 1), perceived family financial situation (very well, quite good/average = 0; quite bad/very bad = 1), physical activity (≥2 times/week = 0; ≤1 time/week = 1; being physically active ≥2 times/week is suggested to reducing insomnia symptoms among adults [21]), school start time (08:00–08:15 = 0; 08.30 or later = 1), cigarette use (<1 time/month = 0; ≥1 time/month = 1), alcohol (<1 time/month = 0; ≥1 time/month = 1), and screen time (defined as social media and/or gaming/TV/film on schooldays; <4 h/school day = 0; ≥4 h/school day = 1; the cut-off score of ≥4 h screen time is based on the assumption that sedentary behavior of ≥4 h a day is associated with insomnia among adolescents [22]). 

Lastly, we used logistic regression analysis to examine the associations between the dependent variable of insomnia (MISS ≥ 6) and the same independent variables as in the bivariate analyses. However, due to multicollinearity, use of alcohol and cigarettes were merged into one variable: cigarette and/or alcohol use (<1 time/month = 0; ≥1 time/month = 1). As physical activity and self-reported health have been found to interact [23], a new variable was also created for the combined effects of physical activity and self-reported health. The variable physical activity ≥2 times/week and good self-reported health was used as reference category, with the following dummy variables: physical activity ≤1 time/week and good self-reported health; physical activity ≥2 times/week and poor self-reported health; and physical activity ≤1 time/week and poor self-reported health.

Model fit was assessed with the Hosmer–Lemeshow goodness-of-fit test, which tests differences between actual and predicted values of the dependent item. Good model fit is indicated by a non-significant *p*-value, indicating no difference between observed and predicted dependent values [24]. Multicollinearity was assessed by tolerance, which should be >0.4 [25]. Nagelkerke’s pseudo-*R^2^* was used to indicate how well the model accounted for the odds of experiencing insomnia according to MISS [26]. IBM SPSS v. 26 (IBM Corp., Armonk, NY, USA) was used for the statistical analyses, and the level of significance was set at *p* < 0.05.

## 3. Results

Of the 1504 participating adolescents, 1477 (98%; 56% females) completed all three MISS items and were thus included in the analysis. Over 90% of the participants perceived their family financial situation as being good or average, and two-thirds (67%) of respondents declared their self-reported health to be good or very good. Insomnia was reported in 22% of the study group, (18% of females and 27% of males), and the median age was 16 years (range 15–17 years).

The bivariate analysis (Table 1) showed that insomnia was associated with being male, a poorer perceived family financial situation, having poor self-reported health, infrequent physical activity (≤1 time/week), having failed at least one school course, and a monthly use of alcohol and/or cigarettes (*p* < 0.05). However, school start time before or after 08:30, and time spent on social media and/or gaming ≥4 h/day were not associated with insomnia (Table 1).

The logistic regression analysis showed that insomnia was associated with being male (OR: 1.4), poor self-reported health (OR: 4.2), failed school courses (OR: 1.4), and monthly use of alcohol and/or cigarettes (OR: 1.4). There was no significant association between insomnia and school start time, perceived family financial situation, physical activity, or spending ≥4 h/day on social media and/or gaming (Table 2).

We then investigated possible combined effects between self-reported health and physical activity on insomnia (Table 3). In this analysis, insomnia was associated with failed school courses (OR: 1.6), monthly use of alcohol and/or cigarettes (OR: 2.1), infrequent physical activity (≤1 time/week) and good self-reported health (OR: 1.7), physical activity (≥2 times/week) and poor self-reported health (OR: 11.9), and infrequent physical activity (≤1 time/week) and poor self-reported health (OR: 18.9).

## 4. Discussion

The aim of the study was to investigate the association between insomnia, academic performance, self-reported health, physical activity, school start time, and substance use among adolescents. Our results show that insomnia was present in 22% of the participants, and that it was more common in males than in females. Further, poor self-reported health, failed school courses, and monthly alcohol or tobacco use were also associated with insomnia in Swedish adolescents. When the combination between self-reported health and physical activity was tested, physical inactivity was also found to be associated with insomnia, and the effect of the combination was strikingly greater than either factor alone.

In the present study, poor self-reported health was correlated with insomnia. Similarly, Roberts et al. [9] demonstrated that insomnia is correlated with future health. Insomnia increases the risk for health problems such as anxiety and depression [9,27]. Insomnia may also come from mental problems such as depression and anxiety disorders, and, therefore, interventions targeting both sleep hygiene and psychological aspects are recommended [28]. Adolescents with insomnia are more likely to seek medical care in the future [9]. 

In this study, insomnia was more common among males than among females. However, females were also more physically active than males in this study; thus, it is likely that physical activity—rather than gender—affected insomnia. In other words, inactive adolescents with poor self-reported health are eighteen times more likely to have insomnia relative to active adolescents with good self-reported health. The World Health Organization estimates that 1.9 million deaths worldwide that are related to chronic disease can be attributed to physical inactivity so it is important to promote active lifestyles [29]. Physical activity may be used as an effective non-pharmacological intervention for improving sleep quality [11]. A meta-analytic review showed that physical activity had positive effects on sleep duration and sleep quality over time [30]. However, a systematic review [31] contended that exercise must be further evaluated to judge its usefulness in the treatment of insomnia. It is unknown what kind of exercise or what “dose” may be most efficient. 

Furthermore, our results revealed that failed school courses were associated with insomnia. Similarly, Zhao et al. [32] reported associations between poor school performance and insomnia among adolescents aged 11–20 years. Worries about failing courses might lead to insomnia. However, given that sufficient sleep is crucial for cognitive functioning and memory consolidation, insomnia might also lead to poor academic performance or both issues may be due to other health problems, such as depression, that may impact both sleep and cognitive performance [33].

Another finding was that the monthly use of alcohol and/or cigarettes was associated with insomnia. Similarly, a study of Japanese adolescents showed that insomnia is prevalent and associated with multiple factors, such as poor mental health, monthly alcohol consumption, and smoking [34]; and another study among adolescents (aged 10–21 years) in North America also reported correlations between substance use and sleep disturbances [35]. Poor sleep has been found to be a predictor of substance use among adolescents [16]. Understanding the association between reducing adolescents’ substance use and its effects on insomnia is important for informing the best ways to promote sleep health in this population. A bidirectional relationship between substance use and insomnia has been proposed in which sleepy adolescents may use nicotine to become more alert during the days and consume alcohol in the evenings to facilitate sleep. Both nicotine and alcohol have, however, sleep disturbing effects and thus vicious cycles are maintained [36]. 

School start time was not associated with insomnia in the present study. Delayed school start time by one hour has been found to be associated with improved alertness, mood, and health among adolescents in a study by Lufti et al. [37]. A Cochrane review regarding high school students showed that a number of school systems worldwide have proposed and implemented later school start time, and there are potential associations between later school start time, academic performance, quality of sleep, and mental health [38]. No studies have been conducted in Sweden, and further studies with a long-term follow up is therefore called for.

The results of the present study can serve as a stepping stone for future research on how society can increase the health and well-being of adolescents experiencing insomnia. Engagement and motivation are crucial aspects that must be addressed in order to change sleep-related behaviors in adolescents. One approach for doing so is the use of motivational interviews, which was found to be effective at extending adolescents’ sleep duration [39]. Parent-set bedtimes have also been found to be beneficial for teenage sleep [40]. Adolescents, guardians, schools, and society in general must adopt a health-promotion framework that includes listening to the adolescents’ thoughts and concerns. Nonetheless, adolescents are well-aware of the importance of adequate sleep [41,42], yet more work remains to be done to motivate adolescents to establish healthy sleep habits. 

### Strengths and Limitations

The strengths of the current study include the large sample size. However, one limitation of self-reported data is that answers can vary from day to day according to external and internal circumstances. Given that the questionnaire was completed during the school day, it may be that adolescents viewed that a completion of the study as mandatory, regardless of the fact that they were clearly informed that a participation in the study was voluntary. Cross-sectional designs cannot determine causal relationships [43]. Other limitations are that questions regarding anxiety or daytime tiredness were not included, and that some of the included questions were single items. The items were dichotomized, which can increase the risk of overlooking nuances in the result; however, this was done as a result of low response frequencies on some response alternatives, and also to facilitate data interpretation.

## 5. Conclusions

Insomnia was associated with poor self-reported health, failed school courses, and substance use. When the combined effect of self-reported health and physical activity were investigated, a combination of low physical activity and poor self-reported health was strongly associated with insomnia among adolescents. This highlights the need for a comprehensive approach to promote health by considering as many sleep-related factors as possible beyond simply insomnia, such as physical activity, academic performance, and substance use. 

## Figures and Tables

**Table 1:** Associations between insomnia (MISS ≥ 6) and other factors among adolescents aged 15–17 years (n = 1477).

Factors	Total	No Insomnia (MISS < 6)	Insomnia (MISS ≥ 6)	*p*-Value ^a^
**Sex, n (%)**				
Female	816 (56.6)	667 (59.0)	149 (47.0)	<0.0001
Male	636 (43.4)	466 (41.0)	170 (53.0)	
**Perceived family financial situation, n (%)**				
Very well/quite good/average	367 (77.1)	1108 (97.8)	298 (94.6)	0.004
Quite bad/very bad	42 (2.9)	42 (2.2)	17 (5.4)	
**Self-reported health, n (%)**				
Very well/good	987 (68.0)	872 (76.5)	115 (36.1)	<0.0001
Neither good nor bad/quite bad/very bad	472 (32.0)	268 (23.5)	204 (63.9)	
**School start, n (%)**				
08:00–08:15	850 (57.6)	674 (58.5)	176 (54.5)	0.202
08:30 or later	626 (42.4)	479 (41.5)	147 (45.5)	
**Physical activity, n (%)**				
≥2 times/week	1001 (68.0)	815 (70.9)	186 (57.9)	<0.0001
≤1 time/week	470 (32.0)	335 (29.1)	135 (42.1)	
**Failed school courses, n (%)**				
None	1254 (85.1)	1004 (87.2)	250 (77.6)	<0.0001
≥1	219 (14.9)	147 (12.8)	72 (22.4)	
**Alcohol use, n (%)**				
<1 time/month	1004 (68.4)	809 (70.5)	195 (61.0)	<0.0001
≥1 time/month	463 (31.6)	339 (29.5)	124 (39.0)	
**Cigarette use, n (%)**				
<1 time/month	1326 (90.3)	1058 (92.0)	268 (84.0)	<0.0001
≥1 time/month	143 (9.7)	92 (8.0)	51 (16.0)	
**Screen time (not school related), n (%) ^b^**				
Screen time <4 h/day	819 (56.0)	665 (58.0)	154 (49.0)	0.072
Screen time ≥4 h/day	636 (44.0)	484 (42.0)	152 (51.2)	

MISS = Minimal Insomnia Symptoms Scale. ^a^ Chi^2^ test. ^b^ Time spent on social media/gaming/TV/film on schooldays.

**Table 2:** Multiple logistic regression analysis of factors associated with insomnia (MISS ≥ 6) among adolescents aged 15–17 years (n = 1477).

Independent Variables	Wald	OR	95% CI for OR	*p*
Male sex	4.692	1.380	1.031–1.846	0.030
Poor self-reported health ^a^	97.515	4.349	3.248–5.822	<0.0001
≥1 failed school course(s)	4.331	1.474	1.023–2.125	0.037
Poor perceived family financial situation	0.460	1.247	0.659–2.359	0.498
Physical activity ≤1 time/week	3.527	1.334	0.987–1.803	0.060
School start at 08:15 or earlier	0.537	0.895	0.665–1.205	0.464
Alcohol and/or cigarette use ≥1 time/month	5.681	1.433	1.066–1.926	0.017
Screen time (not school related) <4 h/day ^b^	0.137	0.947	0.709–1.265	0.711

CI = confidence interval; OR = odds ratio. Hosmer–Lemeshow goodness-of-fit test, *p* = 0.099; Nagelkerke’s pseudo-*R*^2^ = 0.271; there were no signs of multicollinearity (tolerance: 0.7–0.8). ^a^ Neither good nor bad/quite bad/very bad health. ^b^ Time spent on social media/gaming/TV/film on schooldays.

**Table 3:** Multiple logistic regression analysis of combined effects of factors associated with insomnia (MISS ≥ 6) among adolescents aged 15–17 years (n = 1477).

Independent Variables	Wald	OR	95% CI for OR	*p*
Male sex	1.931	0.771	0.534–1.113	0.165
≥1 failed school course(s)	4.384	1.648	0.1674–0.717	0.036
Poor family financial situation	0.442	1.156	0.755–1.770	0.506
School start at 08:15 or earlier	0.093	1.059	0.733–1.530	0.760
Alcohol and/or cigarette use ≥1 time/month	3.850	2.064	1.381–3.084	<0.0001
Screen time (not school related) <4 h/day ^a^	1.473	0.796	0.550–1.151	0.225
Physical activity ≥2 times/week and good health (ref. category)				
Physical activity ≤1 time/week and good self-reported health	6.342	1.752	1.132–2.710	0.012
Physical activity ≥2 times/week and poor self-reported health ^b^	68.106	11.929	6.621–21.493	<0.0001
Physical activity ≤1 time /week and poor self-reported health ^b^	85.111	18.873	10.111–35.227	<0.0001

CI = confidence interval; OR = odds ratio. Hosmer–Lemeshow goodness-of-fit test, *p* = 0.042; Nagelkerke’s pseudo-*R*^2^ = 0.268; there were no signs of multicollinearity (tolerance: 0.7–0.8). ^a^ Time spent on social media/gaming/TV/film on schooldays. ^b^ Neither good nor bad/quite bad/very bad self-reported health.

## Abbreviations

CI:	confidence interval;
MISS:	Minimal Insomnia Symptom Scale;
OR:	odds ratio
